# Correction: Smoking is associated with increased risk of cardiovascular events, disease severity, and mortality among patients hospitalized for SARS-CoV-2 infections

**DOI:** 10.1371/journal.pone.0318361

**Published:** 2025-01-24

**Authors:** Ram Poudel, Lori B. Daniels, Andrew P. DeFilippis, Naomi M. Hamburg, Yosef Khan, Rachel J. Keith, Revanthy Sampath Kumar, Andrew C. Stokes, Rose Marie Robertson, Aruni Bhatnagar

The eighth author’s name is spelled incorrectly. The correct name is: Andrew C. Stokes.
