# Supplementary material for: Smoking is associated with increased risk of cardiovascular events, disease severity, and mortality among patients hospitalized for SARS-CoV-2 infections
Source: PLoS One. 2022 Jul 15;17(7):e0270763. doi: 10.1371/journal.pone.0270763 (PMC9286231; doi:10.1371/journal.pone.0270763)
Supplement: S1 File — (DOCX) [file pone.0270763.s001.docx]

**Supplemental Table 1.** Demographic characteristics of hospitalized adults with SARS-COV-2 infection of the AHA COVID-19 CVD Registry from December 2020 to March 2021 stratified by smoking status prior to propensity matching.

|  | **Overall** | **Non-Smokers** | **Smokers** |
| --- | --- | --- | --- |
|  | **(N=31,545)** | **(N=29,306)** | **(N=2,239)** |
| Patient’s Status |  |  |  |
| Death, n (%) | 4,651 (14.7) | 4,295 (14.7) | 356 (15.9) |
| In Mechanical Ventilator, n (%) | 5,558 (17.6) | 5,132 (17.5) | 426 (19.0) |
| Age (years) |  |  |  |
| Mean (SD) | 62.1 (17.8) | 62.3 (17.9) | 60.0 (16.8)* |
| Median [Min, Max] | 64.0 [18.0, 103] | 62.0 [18.0, 99.0] | 63.0 [18.0, 103.0] |
| Sex |  |  |  |
| Male, n (%) | 16,945 (53.7) | 15,562 (53.1) | 1,383 (61.8)* |
| Female, n (%) | 14,600 (46.3) | 13,744 (46.9) | 856 (38.2)* |
| Race/Ethnicity |  |  |  |
| NH-White, n (%) | 14,527 (46.1) | 13,385 (45.7) | 1,142 (51.0)* |
| Black, n (%) | 7,219 (22.9) | 6,611 (22.6) | 608 (27.2)* |
| Hispanic, n (%) | 6,809 (21.6) | 6,502 (22.2) | 307 (13.7)* |
| Asian/Pacific Islanders, n (%) | 1,236 (3.9) | 1,173 (4.0) | 63 (2.8)* |
| Other, n (%) | 1,754 (5.6) | 1,635 (5.6) | 119 (5.3) |
| Medical History |  |  |  |
| Obesity, n (%) | 15,057 (47.7) | 14,104 (48.1) | 953 (42.6)* |
| Diabetes mellitus, n (%) | 1,882 (37.7) | 11,083 (37.8) | 799 (35.7)* |
| Hypertension, n (%) | 20,852 (66.1) | 19,322 (65.9) | 1,530 (68.3)* |
| Dyslipidemia, n (%) | 15,453 (49.0) | 14,311 (48.8) | 1,142 (51.0)* |
| Deep venous/pulmonary embolus, n (%) | 1,670 (5.3) | 1,525 (5.2) | 145 (6.5)* |
| Coronary artery disease, n (%) | 3,423 (10.9) | 3,068 (10.5) | 355 (15.9)* |
| Peripheral artery disease, n (%) | 890 (2.8) | 780 (2.7) | 110 (4.9)* |
| Cerebrovascular disease, n (%)  Heart Failure, n (%) | 3,627 (11.5)  3,909 (12.4) | 3,368 (11.5)  3,537 (12.1) | 259(11.6)  372 (16.6)* |
| Chronic kidney disease, n (%) | 4,495 (14.2) | 4,144 (14.1) | 351 (15.7)* |
| Medications Use |  |  |  |
| Anti-platelet therapy, n (%) | 9,000 (28.5) | 8,250 (28.2) | 750 (33.5)* |
| Anti-coagulant, n (%) | 4,324 (13.7) | 286 (12.8) | 4,324 (13.7) |
| Time of Admission |  |  |  |
| First quarter, 2020, n (%) | 5,661 (17.9) | 5,286 (18.0) | 375 (16.7) |
| Second quarter, 2020, n (%) | 11,423 (36.2) | 10,679 (36.4) | 744 (33.2)* |
| Third quarter, 2020, n (%) | 4,607 (14.6) | 4,231 (14.4) | 376 (16.8)* |
| Fourth quarter, 2020, n (%)  First quarter, 2021, n (%) | 8,800 (27.9)  1,021 (3.2) | 8,143 (27.8)  935 (3.2) | 657 (29.3)  86 (3.8) |
|  |  |  |  |

NH: Non-Hispanic; SD: Standard deviation

* P<0.05 vs smokers

**Supplemental Table 2**. Characteristics of the propensity-matched study population of the AHA COVID-19 CVD Registry from December 2020 to March 2021 by mechanical ventilator use.

|  | **Overall** | **No Mechanical Ventilator Use** | **Mechanical Ventilator Use** |
| --- | --- | --- | --- |
|  | **(N=6,717)** | **(N=5,535)** | **(N=1,182)** |
| Smoking Status |  |  |  |
| Smokers, n (%) | 2,239 (33.3%) | 1,813 (32.8%) | 426 (36.0%)* |
| Age (years) |  |  |  |
| Mean (SD) | 59.6 (17.8) | 59.4 (18.4) | 60.7 (15.0)* |
| Median [Min, Max] | 61.0 [18.0, 100] | 63.0 [18.0, 93.0] | 71.0 [18.0, 98.0] |
| Sex |  |  |  |
| Male, n (%) | 4,201 (62.5) | 3,382 (61.1) | 819 (69.3)* |
| Female, n (%) | 2,516 (37.5) | 2153 (38.9) | 363 (30.7)* |
| Race/Ethnicity |  |  |  |
| NH-White, n (%) | 3,465 (51.6) | 2,942 (53.2) | 523 (44.2)* |
| Black, n (%) | 1,801 (26.8) | 1,447 (26.1) | 354 (29.9)* |
| Hispanic, n (%) | 922 (13.7) | 742 (13.4) | 180 (15.2) |
| Asian/Pacific Islanders, n (%) | 191 (2.8) | 157 (2.8) | 34 (2.9) |
| Other, n (%) | 338 (5.0) | 247 (4.5) | 91 (7.7)* |
| Medical History |  |  |  |
| Obesity, n (%) | 2,887 (43.0) | 2,320 (41.9) | 567 (48.0)* |
| Diabetes mellitus, n (%) | 2,342 (34.9) | 1,836 (33.2) | 506 (42.8)* |
| Hypertension, n (%) | 4,494 (66.9) | 3,627 (65.5) | 867 (73.4)* |
| Dyslipidemia, n (%) | 3,331 (49.6) | 2,694 (48.7) | 637 (53.9)* |
| Deep venous/pulmonary embolus, n (%) | 413 (6.1) | 344 (6.2) | 69 (5.8) |
| Coronary artery disease, n (%) | 1,025 (15.3) | 834 (15.1) | 191 (16.2) |
| Peripheral artery disease, n (%) | 313 (4.7) | 251 (4.5) | 62 (5.2) |
| Cerebrovascular disease, n (%)  Heart Failure, n (%) | 716 (10.7)  1,049 (15.6) | 593 (10.7)  849 (15.3) | 123 (10.4)  200 (16.9) |
| Chronic kidney disease, n (%) | 992 (14.2) | 784 (14.2) | 208 (17.6)* |
| Medications Use |  |  |  |
| Anti-platelet therapy, n (%) | 2,190 (32.6) | 1,774 (32.1) | 416 (35.2)* |
| Anti-coagulant, n (%) | 810 (12.1) | 650 (11.7) | 160 (13.5) |
| Time of Admission |  |  |  |
| First quarter, 2020, n (%) | 1,142 (17.0) | 815 (14.7) | 327 (27.7)* |
| Second quarter, 2020, n (%) | 2,240 (33.3) | 1,832 (33.1) | 408 (34.5) |
| Third quarter, 2020, n (%) | 1,099 (16.4) | 921 (16.6) | 178 (15.1) |
| Fourth quarter, 2020, n (%)  First quarter, 2021, n (%) | 1,980 (29.5)  252 (3.8) | 1,743 (31.5)  220 (4.0) | 237 (20.1)*  32 (2.7)* |

NH: Non-Hispanic; SD: Standard deviation

* P<0.05 vs mechanical ventilator use
