# Supplementary material for: Smoking is associated with increased risk of cardiovascular events, disease severity, and mortality among patients hospitalized for SARS-CoV-2 infections
Source: PLoS One. 2022 Jul 15;17(7):e0270763. doi: 10.1371/journal.pone.0270763 (PMC9286231; doi:10.1371/journal.pone.0270763)
Supplement: Attachment — Submitted filename: Response to Reviewers.docx [file pone.0270763.s002.docx]

**Response to Reviewers’ Comments**

Reviewer # 1:

1. *“I see the data come from previously published AHA registries. However, did you obtain ethical approval from your committee?”*

Get-With-The-Guidelines® (GWTG) is a continuous quality improvement program registry, overseen by the American Heart Association (AHA) and an Oversight Committee of clinician leaders in quality improvement, that retrospectively collects patient-level adherence data within 3,000 U.S. hospitals. To participate, hospital sites directly and retrospectively abstract or upload quality of care and outcomes data from hospitalized patients’ electronic health records into the registry. Demographic data, clinical data, and test results obtained as part of routine care for CVD patients, as well as pertinent treatments and in-hospital outcomes, are captured. Patient identities (e.g. name, social security number, medical record number, etc.) are not collected. Sites are responsible for assigning a unique study identifier to each submitted record, which is not shared outside the site so the patient cannot be re-identified. The data are collected for quality improvement purposes, and therefore patients are neither recruited nor consented. All research completed using analyses of the AHA GWTG program has been determined to be exempt from IRB oversight by Advarra (see attached). This has been added to the revised text (see page 3).

2. *“Please improve the discussion and the reference section discussing and citing the following PMID”*

Thank you for pointing us to the relevant literature. To the extent possible, we have cited these references both in the introduction and the discussion.

Reviewer # 2:

1. *“The association between smoking and COVID-19 disease severity among people diagnosed with COVID is not "limited" or "unclear." There have been several meta-analyses published…... This language throughout the paper needs to be toned down accordingly.”*

The discussion has been “toned down” as suggested. The “limited” and “unclear” qualifications have been removed, although we do note that not all studies have led to consistent results.

2. *“This situation is different from the question of how smoking affects risk of COVID infection which has been more equivocal, as illustrated in Ref 14 of the manuscript. Because the present manuscript deals only with hospitalized patients, it does not provide any information about the relationship between smoking can COVID risk. This distinction needs to be made clear throughout the paper. Beyond making this distinction and commenting on the lack of consensus on the effect of smoking on risk of COVID infection, discussion of this question should be dropped from the paper, including the Discussion section (pages 7 and * because the data and analysis in this paper does not contribute anything to the discussion of smoking and risk of COVID infection.”*

We have removed the discussion of the relationship between smoking and the risk of COVID-19 infection.

3. *“The authors lump e-cigarette use (vaping) in with smoking in their analysis. They need to justify this approach explicitly, both in terms of biology and by doing a sensitivity analysis showing that doing so does not affect the results. The fact that smoking and vaping are combined needs to be indicated in the abstract. Indeed, if smoking and vaping have similar increases in risk, that would be an important result on its own.”*

The number of individuals in our registry who used e-cigarettes is very small – 41 patients reported e-cig only versus 2198 who reported smoking only without e-cigarettes. In our sensitivity analysis, removal of e-cigarette users from the dataset did not significantly affect the odds ratio of in-hospital death or mechanical ventilator use. This has been stated in the revised manuscript.

4. *“The finding that the risks associated with smoking are higher in younger patients is consistent with a recent meta-analysis that reached this conclusion based on data pooled across studies … The fact that the authors found this result in a single dataset is an important contribution of this paper.”*

Thank you for pointing this out. We have cited the paper in our revised manuscript.

5. *“The authors state that the data are available but do not say where it is deposited so that people can access it.”*

The American Heart Association has a strict policy regarding the use and integrity of its Get-With-The-Guidelines® data and is unable to provide public access to the COVID-19 dataset, in full or in part without other agreements in place. However, researchers are able to go to [www.precision.heart.org](http://www.precision.heart.org) , navigate to data, documentation, and COVID-19 to see data documentation, dictionary and coding information and explore the variables, descriptions, source, type, missingness and distribution of the data.
